# A Role for Nonapeptides and Dopamine in Nest-Building Behaviour

**DOI:** 10.1111/jne.12250

**Published:** 2015-01-26

**Authors:** Z J Hall, S D Healy, S L Meddle

**Affiliations:** * School of Biology, University of St AndrewsSt Andrews, UK; † The Roslin Institute, The Royal (Dick) School of Veterinary Studies, The University of EdinburghEaster Bush, UK

**Keywords:** vasotocin, mesotocin, tyrosine hydroxylase, dopamine, nest building

## Abstract

During nest building in zebra finches (*Taeniopygia guttata*), several regions in the social behaviour network and the dopaminergic reward system, which are two neural circuits involved in social behaviour, appear to be active in male and female nest-building finches. Because the nonapeptides, mesotocin and vasotocin and the neurotransmitter, dopamine, play important roles in avian social behaviour, we tested the hypothesis that mesotocinergic-vasotocinergic and dopaminergic neuronal populations in the social behaviour network and dopaminergic reward system, respectively, are active during nest building. We combined immunohistochemistry for Fos (an indirect marker of neuronal activity) and vasotocin, mesotocin or tyrosine hydroxylase on brain tissue from nest-building and non-nest-building male and female zebra finches and compared Fos immunoreactivity in these neuronal populations with the variation in nest-building behaviour. Fos immunoreactivity in all three types of neuronal populations increased with some aspect of nest building: (i) higher immunoreactivity in a mesotocinergic neuronal population of nest-building finches compared to controls; (ii) increased immunoreactivity in the vasotocinergic neuronal populations in relation to the amount of material picked up by nest-building males and the length of time that a male spent in the nest with his mate; and (iii) increased immunoreactivity in a dopaminergic neuronal population in relation to the length of time that a male nest-building finch spent in the nest with his mate. Taken together, these findings provide evidence for a role of the mesotocinergic-vasotocinergic and dopaminergic systems in avian nest building.

Understanding the neurobiology of reproductive behaviour in vertebrates has long been a focus of neuroendocrine research (1). In birds, these studies have typically investigated the production and perception of courtship song (2), affiliation (3), copulation (4) and parental care (5). One key avian reproductive behaviour that has received much less attention is nest-building behaviour.

The current consensus is that two evolutionarily conserved neural circuits, the social behaviour network and dopaminergic reward system, are important for most reproductive behaviour in vertebrates (6). Functionally, the social behaviour network is considered to be involved in the production of courtship, sexual, affiliative and aggressive behaviours (7), whereas the dopaminergic reward system is implicated in the motivation to perform these behaviours (8). Recent studies from our laboratories have revealed that neuronal activity increases in brain regions within both the social behaviour network and dopaminergic reward system in male and female zebra finches during nest building (9).

Many of the brain regions in the social behaviour network and dopaminergic reward system that exhibit elevated neuronal activity with nest-building behaviour are known to contain populations of neurones characterised as using specific signalling molecules to transmit neuronal information to target brain regions (6). In zebra finches, these populations include the vasotocinergic and mesotocinergic parvocellular neuronal populations in the medial bed nucleus of the stria terminalis (BSTm) of the social behaviour network, which release the nonapeptide hormones vasotocin (the avian analogue of arginine vasopressin in mammals) and mesotocin (the avian analogue of oxytocin in mammals), respectively. In addition to releasing these nonapeptides, which may bind to receptors in brain regions including the social behaviour network (10), these neuronal populations also innervate hypothalamic and social behaviour network targets, including the medial preoptic area (10), which exhibits elevated neuronal activity during nest building (9). These populations are distinct from the parvocellular paraventricular neurones forming part of the hypothalamic-pituitary-adrenal stress response axis that are vasotocinergic (11). Central effects on behaviour via dendritic release from magnocellular vasotocin and mesotocin neuronal populations may be predicted to occur in birds as reported in mammals (12–14), although this remains to be established and was not the focus of the present study. In the dopaminergic reward system, dopaminergic neuronal populations in the ventral tegmental area and central grey synthesise and release the neurotransmitter dopamine, which may act on dopaminergic receptors in both the striatum and regions in the social behaviour network, including BSTm and the septum (15,16), amongst other sites.

The actions of the vasotocinergic, mesotocinergic and dopaminergic neuronal populations appear to mediate many reproductive behavioural functions that are enhanced during the breeding season. For example, in zebra finches, vasotocinergic neurones in the BSTm are considered to be involved in affiliative behaviour and courtship (17) and dopaminergic neurones in the central grey appear to be involved in vocal communication with conspecifics (18). Nonapeptidergic signalling has diverse behavioural effects in mammals: oxytocin can suppress appetite; it stimulates female sexual receptivity and male sexual arousal, as well as grooming behaviour, and also is anti-anxiolytic (19), and vasopressin has been implicated in social behaviour, including pair bonding social recognition and aggression (20). These nonapeptides also have a well-established important role in parental behaviour in mammals (19,21–23); for example, i.c.v. oxytocin infusions induce maternal behaviour in rats and mice (23,24) and oxytocin signalling appears to be involved in nest-building behaviour in rodents (25,26). Therefore, we hypothesised that the vasotocinergic, mesotocinergic and dopaminergic neuronal populations within the social behaviour network and dopaminergic reward system are also involved in nest building, a key parental behaviour in birds. In the present study, we compared nest-building behaviour in male and female zebra finches with concurrent neuronal activity, as measured indirectly by the number of neurones producing the immediate early gene protein product Fos (27), in vasotocinergic and mesotocinergic neuronal populations in subdivisions of the BSTm and dopaminergic neuronal populations in the ventral tegmental area and central grey. Because we previously established that neuronal activity in the dorsal subdivision of the BSTm (BSTmd) increased in relation to the length of time that female finches spent in the nest (9) and Klatt and Goodson (28) found that the delivery of a mesotocin antagonist in the zebra finch brain reduces the amount of time nest-building female finches spent in the nest, we hypothesised that mesotocinergic neurones in the ventral subdivision of BSTm (BSTmv) may be involved in the amount of time a female finch spends within the nest and predicted that Fos and mesotocin neurone immunoreactive co-localisation will reflect this brain–behaviour relationship. Neuronal activity also increases in the BSTmd during nest building (9) and so we also predicted that Fos immunoreactivity in vasotocinergic and mesotocinergic neurones in the BSTmd would be higher in nest-building finches compared to controls.

Finally, we aimed to further understand the role of the ventral tegmental area in nest building (9). If increases in neuronal activity involve the dopaminergic neuronal population in this region, then Fos immunoreactivity within dopaminergic neurones in the ventral tegmental area should increase the more often male finches pick up nest material.

## Materials and methods

### Animals

We bred thirty-two adult zebra finches (n = 16 male, n = 16 female) in captivity at the University of St Andrews, St Andrews, Scotland, UK. Prior to experimentation, we housed all birds in single-sex group cages containing 10–20 birds with *ad lib*. access to finch seed mix and water. All birds were maintained under a 14 : 10 h light /dark cycle (lights on 08.00 h) at 19–27 °C and 50–70% relative humidity. All procedures in the present study were performed with permission from the University of St Andrews Animal Welfare and the Ethics Committee and the UK Home Office (PPL. 60/3666).

### Treatment group assignment

We randomly paired zebra finches in opposite-sex pairs in wooden/wire mesh cages (40 × 30 × 39 cm) and housed finch pairs within the same room as the single-sex group cages. Pair cages were fitted with a wooden nest cup (11 × 13 × 12 cm), the floor of the cage was covered with bedding chips, and finch pairs were given access to finch seed mix and water *ad lib*. Finches were paired for a minimum of 1 week before receiving 15-cm lengths of string (No. 4 Polished Cotton Twine; Rope Source, Bolton, UK) as nest material. Prior to receiving string, finch pairs regularly filled their nest cups with bedding chips from the cage floor and some females laid eggs in these bedding nests. We removed bedding and eggs from nest cups daily.

At least 1 week after pairing, we gave four pairs of birds 50 pieces of string at 12.00 h. We inspected cages 24 h later to identify pairs that had begun building a string nest in their nest cup. To create an experimental cohort, we randomly assigned each finch pair that had begun building a nest to one of the two behavioural treatment groups (nest-building and control group). We selected only finch pairs that had begun building a nest to ensure that all pairs included in the present study were motivated and capable of building nests prior to nest building observation.

After allocating birds to treatment groups, we removed all string from the cages of both finch pairs in the experimental cohort and also removed the nest cup from the cage of the control finch pair. We removed the bedding chips from the cages of both pairs to prevent the birds using them for nest building, lined the cage floors with black plastic, and moved both pairs to the experimental room. We repeated this selection procedure until we had eight nest-building and eight control zebra finch pairs.

### Nest building

Once in the experimental room, the control and nest-building pairs were visually but not acoustically isolated from each other by a wooden barrier. To record out-of-nest cup behaviour, we positioned a camcorder in front of each pair's cage (Sony Handycam AVCHD, Model no. HDR-CX115E; Sony Corp., Tokyo, Japan) and, to record in-nest cup behaviour, we suspended a bird-box camera inside each pair's cage (SpyCameraCCTV, Bristol, UK). We left each cohort undisturbed in the experimental room for 24 h to habituate.

Thirty minutes after lights on, and on the morning after habituation, we gave the nest-building pair 250 pieces of string and began filming both pairs. An experimenter observed the birds from outside the test room via a window until the male of the nest-building pair made three consecutive trips with nest material from the cage floor to the nest. We recorded these trips as the time at which the male began to build and sacrificed the birds 90 min later. If the male began building immediately after receiving material, we delayed the start of the observation for 15 min to avoid sampling Fos production in the brain associated with the bird seeing the experimenter.

### Behaviour coding

We encoded the birds’ behaviour using noldus observer (TrackSys Ltd, Nottingham, UK) behavioural analysis software and measured the occurrence of behaviours performed 80–50 min prior to sacrifice, a time bin in which Fos production is associated with these nest-building behaviours (9). Briefly, we measured instances of hopping, feeding, drinking, preening, scratching and allopreening in all birds. In males, we recorded the number of song bouts and the time spent singing (s). In nest-building birds, we measured six nest-building behaviours: pick up (when the bird picked up a piece of string), put down (when the bird deposited a piece of string into the nest), tuck (when the bird used its beak to push a piece of string into the nest when in the nest cup), nest visits and time in nest (s). We also measured time together (s) in the nest (i.e. the duration both members of a nest-building pair spent together in the nest cup).

### Tissue collection

Ninety minutes after the start of nest building, an experimenter entered the room to confirm visually that string was deposited in the nest cup. Once confirmed, we terminally anaesthetised (0.2 ml i.p.; Dolethal; Vétoquinol, Buckinghamshire, United Kingdom) both pairs of birds and rapidly dissected their brains from their skulls. We fixed brains via submersion in 4% paraformaldehyde in phosphate-buffered saline (PBS) (0.1 m; pH 7.4) for 6 days and then moved the brains to 20% sucrose in PBS overnight and then to 30% sucrose in PBS for another night to cryoprotect them. An experimenter removed cerebella from the rest of the brains and froze both the cerebella and remaining brain on pulverised dry ice and stored all neural tissue at −80 °C before transporting the brains on dry ice to the Roslin Institute (University of Edinburgh, Roslin, UK), where the samples were again stored at −80 °C. An experimenter sectioned brains coronally (section thickness = 52 μm) using a freezing microtome and collected sections in four, alternating series in cryoprotectant and stored the sections at −20 °C until free-floating immunohistochemical processing. The cerebella were processed for Fos immunoreactivity in a separate study (Hall Z.J., Ihalainen E., Meddle S.L., Healy S.D., in preparation).

### Double-label immunohistochemistry

Three series of sections were rinsed four times in 0.2% Triton X-100 (Sigma, St Louis, MO, USA) in 0.1 m phosphate buffer (PBT) and once in 0.1 m PBS before being incubated in 0.3% H_2_O_2_ in PBS for 15 min at room temperature to reduce endogenous peroxidase activity. After three PBT rinses, sections were incubated in 10% normal goat serum (Vector Laboratories, Burlingame, CA, USA) in PBT for 60 min at room temperature. Sections were then moved into the primary Fos antibody (rabbit polyclonal anti-Fos K-25, sc-253, Santa Cruz Biotechnology, Santa Cruz, CA, USA, dilution 1 : 10 000) in 10% normal goat serum in PBT and incubated for 21 h at 4 °C. The K-25 Fos antibody has been extensively used in zebra finches (29) and validated in songbirds (30). Sections were then rinsed three times in PBT and incubated in biotinylated goat anti-rabbit secondary antibody (diluted 1 : 250 in PBT; Vector Laboratories) for 1 h at room temperature. After another three rinses in PBT, sections were then incubated in avidin-biotin horseradish-peroxidase complex (Vector Laboratories, dilution 1 : 400) in PBT for 1 h at room temperature. After four rinses in PBT, one rinse in PBS, and a brief rinse in 0.1 m sodium acetate, sections were visualised with 0.04% nickel-intensified diaminobenzidene (Sigma) solution for 210 s at room temperature to develop Fos immunoreactivity and then rinsed five times with PBS to terminate the reaction.

Immediately after Fos visualisation, we double-labelled each series to visualise tyrosine hydroxylase, vasotocin or mesotocin. Tyrosine hydroxylase is an enzyme catalysing the rate-limiting step in dopamine synthesis and is used as a marker for dopaminergic neurones in vertebrate neuroanatomy (6). Briefly, tissue series were rinsed three times in PBT, once in PBS and incubated in 0.3% H_2_0_2_ for 15 min. After another three PBT rinses, tissue series were incubated in blocking serum (tyrosine hydroxylase: 10% normal horse serum, Vector Laboratories; vasotocin and mesotocin: 3% normal goat serum, Vector Laboratories) in PBT for 60 min at room temperature. Sections were then transferred into PBT containing the appropriate blocking serum and primary antibody (tyrosine hydroxylase: mouse monoclonal; Millipore, Billerica, MA, USA, MAB5280, dilution 1 : 1000; vasotocin: rabbit polyclonal; a gift from David A. Gray, University of the Witwatersrand, Johannesburg, South Africa, dilution 1 : 10 000) and incubated for 60 h at 4 °C. Sections destined for mesotocin double-labelling were incubated in primary antibody (mesotocin: rabbit polyclonal; ImmunoStar Inc., Hudson, WI, USA, 20,068, dilution 1 : 5000) for 87 h at 4 °C. All primary antibodies included in the present study have been used and validated previously in birds [tyrosine hydroxylase (31), vasotocin (32,33), mesotocin (34)]. The vasotocin antibody exhibits no cross-reactivity with oxytocin, mesotocin and angiotensin (33) and the mesotocin antibody exhibits no cross-reactivity with vasotocin (34). The specificity of the tyrosine hydroxylase antibody has been extensively addressed by the manufacturer (Millipore; see manufacturer's data sheet) and has wide vertebrate species cross-reactivity including avian species. We also assessed antibody specificity by performing a series of immunohistochemical tests that did not result in any staining and these included complete omission of the primary or secondary antibodies. The distribution of cytoplasmic immunostaining in the zebra finch brain for each primary antibody was as expected compared to the distribution of other published studies using different primary antibodies [tyrosine hydroxylase (35); mesotocin and vasotocin (28)] and also for the known expression of each peptide mRNA. In addition, a dilution series of each primary antibody, when determining the optimum concentration of primary antibody to use, resulted in a correlated reduction in staining intensity. After three further rinses in PBT, tissue was incubated in a solution containing biotinylated secondary antibody (tyrosine hydroxylase: horse anti-mouse, Vector Laboratories, dilution 1 : 100; vasotocin and mesotocin: goat anti-rabbit, Vector Laboratories, dilution 1 : 100) in PBT for 60 min at room temperature. After three rinses in PBT, sections were then incubated in avidin-biotin horseradish-peroxidase complex (Vector Laboratories, dilution 1 : 50) in PBT for 60 min at room temperature. After a final four rinses in PBT and a single rinse in PBS, the second label was visualised by incubating tissue in diaminobenzidene at room temperature for different periods of time depending on the tissue series (tyrosine hydroxylase, 110 s; vasotocin, 225 s; mesotocin, 140 s). Tissue was rinsed five times in PBS to terminate the diaminobenzidene reaction. This labelling procedure produced an intensely dark, black Fos labelled nuclei in neurones and a light brown cytoplasmic staining of neurones producing tyrosine hydroxylase, vasotocin or mesotocin. After double-labelling, all tissue sections were mounted in series on to 0.5% gelatine-subbed microscope slides (Thermo), serially dehydrated in alcohol (70–99%), cleared in xylene and cover-slipped with Pertex mountant (CellPath).

### Quantification of Fos immunoreactivity

We quantified Fos immunoreactivity in neuronal populations characterised by their production of vasotocin, mesotocin or tyrosine hydroxylase. We located each neuronal population with reference to full-section architecture (36) and, more specifically, visualisation of vasotocin, mesotocin and tyrosine hydroxylase. In both vasotocin- and mesotocin-labelled tissue, we sampled vasotocinergic and mesotocinergic populations in the BSTmd in three adjacent sections and BSTmv in two adjacent sections in each brain. We were unable to sample Fos co-localisation in vasotocinergic and mesotocinergic neuronal populations in the supraoptic or paraventricular nuclei because vasotocin and mesotocin immunoreactivity in neurones in both of these regions was too intense to determine whether Fos immunoreactivity was present in the nuclei of these neurones. In tyrosine hydroxylase-labelled tissue, we sampled tyrosine hydroxylase-immunoreactive (dopaminergic) populations in the ventral tegmental area in three adjacent sections and central grey in four adjacent sections in each brain.

In each neuronal population, we counted the number of neurones producing vasotocin, mesotocin or tyrosine hydroxylase and the number of double-labelled (vasotocin + Fos, mesotocin + Fos, or tyrosine hydroxylase + Fos) neurones. Although tyrosine hydroxylase + Fos neurones could be counted in the ventral tegmental area visually using the microscope, single-labelled tyrosine hydroxylase-immunoreactive neuronal populations were too extensive to be quantified using this method. To count these neurones, we took photomicrographs of all ventral tegmental area sections using a × 20 objective lens and counted the tyrosine hydroxylase-immunoreactive neurones using imagej, version 1.45 (NIH, Bethesda, MD, USA). All neurone counts were made in both hemispheres. To account for differences in vasotocinergic, mesotocinergic and dopaminergic neuronal population sizes between sections and birds, we divided the total number of double-labelled cells by the total number of vasotocinergic, mesotocinergic or dopaminergic neurones, respectively, in a given brain to quantify Fos immunoreactivity as the percentage of a neuronal population immunoreactive for Fos.

### Statistical analysis

We used pasw software, version 19.00 (SPSS Inc., Chicago, IL, USA) for all of our statistical analyses. We compared the amount of non-nesting behaviours exhibited by birds using generalised linear model (GLMs) with independent variables including sex on two levels (male and female) and treatment on two levels (nest building and control). We compared the number of song bouts and time spent singing between male control and nest-building birds using the Mann–Whitney *U*-test because these data did not conform to the assumptions of parametric statistical analysis. Similarly, we compared nest-building behaviours between nest-building male and female birds using Mann–Whitney *U*-tests. Finally, we compared Fos immunoreactivity in each neuronal population using GLMs with independent variables including sex on two levels (male and female) and treatment on two levels (nest building and control), as above for non-nesting behaviour.

To investigate whether nest-building behaviour explained individual variation in Fos immunoreactivity, we used multiple linear regression including neuronal activity as a dependent variable and all recorded behaviours in nest-building birds as independent predictors. We ran regression models separately for each sex and each vasotocinergic, mesotocinergic and dopaminergic neurone population sampled using a stepwise reduction procedure to identify behaviours that significantly explained individual differences in Fos immunoreactivity in these populations.

## Results

### Behavioural analysis

Although control and nest-building birds did not differ in how often they hopped, preened, scratched, drank and allopreened (all P* *>* *0.05), control birds fed significantly more often than nest-building birds (F_1,26_ = 3.494, P* *<* *0.001; control: 131.64 ± 13.38 feeds; nest-building: 32.63 ± 12.52 feeds). Control male birds also sang more often (U = 9.0, P* *=* *0.027; control: 14 ± 5.7 song bouts, nest-building: 2.8 ± 0.7 song bouts) and for longer (U = 9.0; P* *=* *0.028; control: 50.7 ± 21.6 song bouts, nest-building: 12.6 ± 4.9 song bouts) than nest-building males. Overall, control birds spent time feeding and, in the case of males, singing, when nest-building opportunities were not available.

Nest-building males picked up (U = 64.0, P* *=* *0.001; male: 143.3 ± 36.5 pick ups, female: 8.8 ± 4.7 pick ups) and deposited (U = 64.0, P* *<* *0.001; male: 68.8 ± 10.5 deposits, female: 0.1 ± 0.1 deposits) nest material significantly more often than nest-building females. Male and female nest-building finches did not differ in the number of times they tucked nest material into the nest structure (U = 48.0, P* *=* *0.093). Nest-building male finches visited the nest cup more often (U = 64.0, P* *=* *0.001; male: 79.1 ± 10.8 visits, female: 16.0 ± 2.9 visits) than nest-building females; however, male and female nest-building finches did not differ in the total amount of time they spent in the nest (U = 36.5, P* *=* *0.473), suggesting that females made fewer but longer trips to the nest cup than their mates.

### Vasotocinergic neuronal populations

Overall, Fos immunoreactivity in vasotocinergic neurone populations in the BSTmd or BSTmv did not differ between nest-building birds and control birds (BSTmd: F_1,26_ = 0.396, P* *=* *0.535; BSTmv: F_1,25_ = 0.001, P* *=* *0.978).

**Figure 1:**
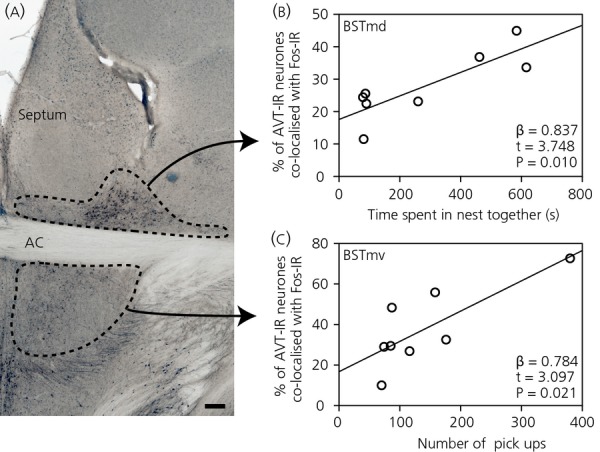
(a) Photomicrograph of medial bed nucleus of the stria terminalis (BSTm) immunocytochemically labelled for arginine vasotocin and Fos with dotted lines indicating the boundaries of vasotocinergic neuronal populations sampled in the present study. Scale bar = 100 μm. (b) Correlation between the time that a pair of nest-building zebra finches spent together in the nest and the percentage of arginine vasotocin immunoreactive (AVT-IR) neurones in the BSTm, dorsal subdivision (BSTmd) immunoreactive for Fos in males. (c) Correlation between the number of times male nest-building zebra finches picked up pieces of nest material and the percentage of vasotocinergic neurones in the BSTm, ventral subdivision (BSTmv) immunoreactive for Fos in males. AC, anterior commissure.

Among nest-building males, Fos immunoreactivity in vasotocinergic neurones in the BSTmd increased in relation to the length of time a male spent together with his mate in the nest cup (β = 0.837; t = 3.748; F_1,6_ = 14.048; P* *=* *0.010) (Fig.1). Additionally, Fos immunoreactivity in vasotocinergic neurones in the BSTmv increased the more often males picked up pieces of nest material (β = 0.784; t = 3.097; F_1,6_ = 9.590; P* *=* *0.021) (Fig.1). In the female mates of nest-building males, none of the behaviours that we measured significantly explained the individual variation in Fos immunoreactivity in vasotocinergic populations in either BSTmd or BSTmv.

### Mesotocinergic neuronal populations

**Figure 2:**
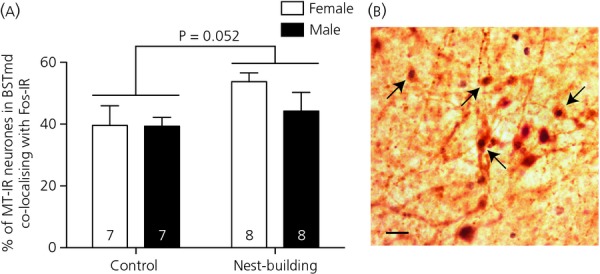
(a) Fos immunoreactivity in mesotocin-immunoreactive (MT-IR) neurones in the medial bed nucleus of the stria terminalis, dorsal subdivision (BSTmd) in adult control and nest-building zebra finches. Bars represent mean + SEM percentage of MT-IR neurones immunoreactive for Fos in BSTmd in female (white bars) and male (black bars) zebra finches of pairs in which the male was or was not constructing a nest . The n value for each group is printed within each bar on the graph. (b) A photomicrograph of neurones in the BSTmd immunoreactive for MT (cytoplasmic brown stain) and Fos (black nuclear stain) in a male nest-building zebra finch. Scale bar = 20 μm. Arrows indicate examples of double-labelled cells.

Fos immunoreactivity in mesotocinergic neurones in the BSTmd (but not in the BSTmv) tended to be greater in nest-building birds than in controls (BSTmd: F_1,26_ = 4.160, P = 0.052; BSTmv: F_1,25_ = 0.612; P* *=* *0.441) (Fig.2).

None of the behaviours that we measured significantly explained individual variation in Fos immunoreactivity in mesotocinergic neurones in either BSTmd or BSTmv in nest-building males or females.

### Dopaminergic neuronal populations

Overall, Fos immunoreactivity in dopaminergic neurones in both the ventral tegmental area and central grey did not differ between the nest-building and control birds (ventral tegmental area: F_1,26_ = 0.488; P* *=* *0.491; central grey: F_1,26_ = 2.880; P* *=* *0.102).

**Figure 3:**
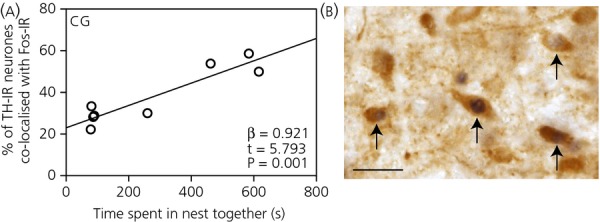
(a) Correlation between the time a pair of nest-building zebra finches spent together in the nest cup and Fos immunoreactivity in tyrosine hydroxylase-immunoreactive (TH-IR) neurones in the central grey (CG) of male zebra finches. (b) A photomicrograph of neurones labelled for TH (cytoplasmic brown stain) and Fos (black nuclear stain) immunoreactivity. Scale bar = 20 μm. Arrows indicate neurones containing both labels.

Among nest-building males, Fos immunoreactivity in dopaminergic neurones in the central grey increased in relation to the length of time a male spent with his mate in the nest cup (β = 0.921; t = 5.793; F_1,6_ = 33.564; P* *=* *0.001) (Fig.3). Additionally, Fos immunoreactivity in dopaminergic neurones in the ventral tegmental area decreased in relation to the amount of nest material males tucked into the nest (β = −0.719; t = −2.531; F_1,6_ = 6.405; P* *=* *0.045).

In females paired with nest-building males, Fos immunoreactivity in dopaminergic neurones in the ventral tegmental area decreased in relation to the amount a female fed (β = −0.816; t = −3.453; F_1,26_ = 11.923; P* *=* *0.014). None of the behaviours that we measured significantly explained individual variation in Fos immunoreactivity in dopaminergic neurones in the central grey of female nesting finches.

## Discussion

### Vasotocinergic and mesotocinergic neuronal populations

#### Nonapeptide hormones and parental behaviour

The demonstration that vasotocinergic and mesotocinergic neuronal populations are active during nest building in zebra finches suggests that, in addition to nonapeptide hormones acting in the brain to regulate pair formation (37), they may also be involved in nest building. In conjunction with previous studies demonstrating a role for nonapeptide signalling within the brain in parental behaviour in mammals (38) and fish (39), it appears that a role for nonapeptide hormone systems in parental behaviour may be evolutionarily conserved across vertebrate taxa. As recently highlighted by Kelly and Goodson (17), correlational studies demonstrating a relationship between neuronal activity in nonapeptidergic neuronal populations and social behaviours require complementary studies in which neuronal activity in these cells is manipulated within specific cell groups to establish a functional connection between brain and behaviour. It should also be noted that, because we used a relatively limited sample size and because Fos immunohistochemistry suffers from a lack of temporal specificity in relation to specific patterns of neuronal activity, the lack of a correlation between Fos immunoreactivity in a given neuronal population and a specific behaviour should not be used as evidence to preclude the involvement of that brain region in the behaviour of interest because some brain–behaviour relationships may not be detected using Fos immunohistochemistry or a smaller sample size.

#### BSTmd

In the present study, we found that the previously-reported increase in BSTmd Fos immunoreactivity in nest-building finches compared to nonbuilding controls (9) may occur specifically within mesotocinergic neurones. Because this increase in immunoreactivity did not correlate with any nest-building behaviour, it may be related to nest possession or perception of the nest rather than to nest-building behaviour *per se*, which is consistent with the findings reported by Hall *et al*. (9). This possibility requires explicit testing. The limited power in this group difference reported by Hall *et al*. (9) and the present study suggests that this group difference may be better tested with a larger sample size in the future.

Within the BSTmd, Fos immunoreactivity in vasotocinergic neurones also increased in male nest-building finches in relation to the length of time a male spent together with his mate in the nest, which appears to be at odds with the absence of a relationship between Fos immunoreactivity in vasotocinergic neurones in the BSTm and the time spent in the nest in zebra finches (28). This discrepancy may be explained in part by differences in quantifying BSTmd and BSTmv Fos immunoreactivity separately or together (9) and the behaviours measured. Although Klatt and Goodson (28) and Hall *et al*. (9) measured the amount of time individual birds spent within the nest, in the present study, we measured the amount of time the pair of finches spent together in the nest. This finding might be particularly important because vasotocinergic neurones in the BSTm appear to be involved in eliciting affiliative responses to mates (40). These results support the hypothesis that vasotocinergic neuronal populations in BSTm of male birds may be involved in affiliative behaviour (17), including social interactions within the nest during nest building, although further studies are necessary to test this possibility.

#### BSTmv

Although Fos immunoreactivity in the BSTmv was unrelated to nest-building behaviour in male finches in a previous study (9), in the present study, we found that Fos immunoreactivity in vasotocinergic neurones increased in the BSTmv in relation to the amount of time that a nest-building male finch picked up nest material. This difference may be explained if the relationship between neuronal activity and picking up nest material is specific to vasotocinergic neurones in this region. The earlier result (9) may have been the result of a masking of the total Fos immunoreactivity in other neuronal populations located within the BSTmv. Functionally, vasotocinergic neurones in the BSTmv of zebra finches may be involved in picking up nest material. As antagonising vasotocin signalling in the brain did not affect nest material collection behaviour by male nest-building zebra finches (28), it is plausible that vasotocinergic neurones in the BSTmv influence nest material collection via their neuronal activity. It should be noted that the number of birds contributing to this relationship is limited and would benefit from replication using large sample sizes or manipulations to test for a functional relationship between the BSTmv and nest material collection.

The lack of a relationship between Fos immunoreactivity in either vasotocinergic or mesotocinergic neuronal populations and the time that a female spent in the nest suggests that other neuronal populations located in the BSTmv intermingled with the nonapeptidergic populations sampled in the present study, such as the population of neurones expressing receptors for vasoactive intestinal peptide (41), may increase their activity in relation to the length of time a female finch spends in the nest. This vasoactive intestinal peptide-sensitive neuronal population may be particularly interesting in the context of nest building because vasoactive intestinal peptide signalling regulates prolactin release, which is involved in maternal behaviour including incubation in birds (42). The involvement of another neuronal population aside from vasotocinergic and mesotocinergic neurones in the relationship between neuronal activity in BSTmv and time spent in the nest also explains why central infusions of pharmacological antagonists that impair vasotocin and mesotocin signalling did not affect the time female zebra finches spent within the nest (28).

### Dopaminergic neuronal populations

#### Ventral tegmental area

Fos immunoreactivity in dopaminergic neurones within the ventral tegmental area was not correlated with nest material collection by male finches, and so it appears that this dopaminergic neuronal population does not play a role in collecting nest material. The negative correlation between Fos immunoreactivity in the ventral tegmental area dopaminergic neurones and the the number of times male finches tucked pieces of nest material into the nest structure may instead suggest that tucking nest material into the nest structure is unrewarding or that the dopaminergic neurone population in the ventral tegmental area inhibits tucking behaviour in male finches. Pharmacological manipulations could be used to inhibit neuronal activity in ventral tegmental area dopaminergic neurones to distinguish between these two possibilities.

Banerjee *et al*. (43) found that Fos immunoreactivity increased in dopaminergic neurones in the ventral tegmental area in recently paired male and female zebra finches, who were also observed to spent more time in the nest together than controls. In the present study, we found no relationship between time spent in the nest together with Fos immunoreactivity in dopaminergic neurones in the ventral tegmental area; however, we also waited for a minimum of 1 week after pairing to record nest-building behaviour, suggesting that this increased neuronal activity may be specific to recently-paired birds.

Finally, in female nest-building finches, we found that Fos immunoreactivity in dopaminergic neurones in the ventral tegmental area decreased in relation to how often these females fed. This negative relationship contrasts with the well-known relationship that dopamine has in the ventral tegmental area mediating food rewards (44). It should be noted that this negative relationship only appeared in our nest-building female finches and studies dedicated to identifying neural substrates of feeding in birds are required to begin to clarify the biological significance of this result.

#### Central grey

The increase in Fos immunoreactivity in central grey dopaminergic neurones in male nest-building finches the more time they spent in the nest with their partners supports the proposal that dopaminergic neurones in the central grey may play a role in social communication (3) between a male finch and his female partner. Consistent with the central grey playing a role in pair interaction during nest building specifically, Banerjee *et al*. (43) found that Fos immunoreactivity increased in dopaminergic neurones in the central grey of male finches that had recently been paired with a female and spent more time in the nest compared to controls. This social communication might take the form of ‘duet-like’ vocalisations that appear to be performed during nest building (45) but, as yet, we have no data to confirm this possibility.

Taken together, these data provide the first evidence that vasotocinergic, mesotocinergic and dopaminergic neuronal populations in the social behaviour network and dopaminergic reward system are active when birds are nest building. These brain–behaviour relationships suggest that nest-building behaviour can be classified as a social behaviour regulated by the social behaviour network and dopaminergic reward system and also provide a robust neuroendocrinological foundation for future studies on the neurobiology of nest-building behaviour. Furthermore, these data support the suggestion that nonapeptidergic systems in the brain play an evolutionarily conserved role in controlling parental behaviour in vertebrates, including nest building in birds.
